# Analyzing safety and effectiveness of Mavacamten in comparison with placebo for managing hypertrophic cardiomyopathy: a systemic review and meta-analysis

**DOI:** 10.1186/s43044-023-00427-5

**Published:** 2023-12-02

**Authors:** Hussain Sohail Rangwala, Hareer Fatima, Mirha Ali, Syed Taha Ahmed, Burhanuddin Sohail Rangwala, Syed Raza Abbas

**Affiliations:** 1 Department of Medicine, Jinnah Sindh Medical University, Iqbal Shaheed Rd., Karachi, Pakistan; 2 Department of Medicine, Dow University of Health Sciences, Karachi, Pakistan

**Keywords:** Hypertrophic cardiomyopathy, Mavacamten, Systemic review, Meta-analysis

## Abstract

**Background:**

Hypertrophic cardiomyopathy (HCM) is a hereditary myocardial disorder, often due to sarcomere gene mutations, characterized by the left ventricular hypertrophy. Current treatments offer symptomatic relief but lack specificity. Mavacamten, an allosteric inhibitor, has shown significant improvements in HCM patients in trials, reducing the requirement for invasive treatments. This meta-analysis assesses Mavacamten’s efficacy and safety as a targeted HCM intervention.

**Methods:**

This study examined four randomized controlled trials comparing Mavacamten to placebo in HCM patients. Each trial had a unique primary endpoint, and secondary outcomes included improvements in NYHA-FC, eligibility for septal reduction therapy (SRT) or undergoing it, adverse events (serious and treatment-related), atrial fibrillation, and non-sustained ventricular tachycardia. Statistical analysis involved calculating risk ratios (RRs) and assessing heterogeneity.

**Results:**

The four included studies showed minimal risk of bias and involved 503 patients with HCM (273 Mavacamten and 230 placebo). Mavacamten significantly increased the primary endpoint (RR 2.15, 95% CI 1.20–3.86, *P* = 0.01) and ≥ 1 NYHA-FC class (RR 2.21, 95% CI 1.48–3.3, *P* = 0.0001). Mavacamten group had lower rates of SRT compared to those receiving placebo (RR, 0.30, 95% CI 0.22–0.40; *P* < 0.00001). No significant differences existed in rates adverse events between the Mavacamten and placebo groups.

**Conclusions:**

Our study suggests that Mavacamten may have therapeutic benefits for HCM patients, as indicated by its positive impact on certain endpoints. Further research with larger samples, longer follow-up, and comprehensive analysis is needed to understand Mavacamten’s safety and efficacy in HCM patients.

## Background

Hypertrophic cardiomyopathy (HCM) is a prevalent hereditary condition triggered by sarcomere gene mutation. It is distinguished by an autosomal dominant inheritance pattern. This myocardial disorder clinically presents with the left ventricular (LV) hypertrophy, primarily attributed to genetic variants affecting sarcomere-encoding genes [1–4]. HCM can be categorized into two primary groups: obstructive HCM (oHCM), which constitutes 70% of most cases. It is distinguished by the obstruction of the left ventricular outflow tract (LVOT), characterized by a gradient of 30 mm Hg or higher at rest or under excitation. Conversely, non-obstructive HCM (nHCM) represents the remaining cases and is defined by the absence of significant LVOT obstruction, with gradients measuring less than 30 mm Hg at rest or with excitation [5, 6]. Regardless of the hemodynamic status, mutations in sarcomere genes initiate an excessive interaction between cardiac actin and myosin, resulting in hypercontractility. This, in turn, leads to decreased relaxation of the myocardium and abnormal compliance, which are distinctive features of this condition [3].

The present management approach for HCM primarily revolves around alleviating symptoms, evaluating the possibility of sudden cardiac death, implementing preventive measures, and conducting family screenings [6]. Symptomatic treatment approach entails using beta-blockers and non-dihydropyridine calcium channel blockers to alleviate obstruction. However, current pharmacological treatments for oHCM offer symptomatic relief without addressing the fundamental pathophysiological mechanisms underlying HCM or modifying the disease's progression [2, 6–9]. While these therapies effectively improve symptoms in most patients, they lack specificity for HCM and fail to target the underlying biochemical abnormalities associated with the condition [10]. Septal reduction therapy (SRT), encompassing both alcohol septal ablation and surgical septal myectomy, stands as the benchmark for alleviating symptoms that remain unresponsive to medical treatment, primarily when these symptoms significantly impair one's quality of life [6, 10–12]. It is crucial to remember that SRT requires intrusive treatments and carries some hazards. Additionally, not every location may have easy access to the knowledge needed to execute SRT [13, 14].

Mavacamten, an innovative and pioneering allosteric inhibitor of β-cardiac myosin, is a first-in-class medication that selectively reversibly disrupts the bond between cardiac actin and myosin, effectively reducing the formation of actin–myosin cross-bridges. This unique mechanism of action directly targets the fundamental pathophysiological mechanism underlying oHCM, leading to a significant decrease in myocardium contractility and ventricular compliance [15, 16]. The remarkable efficacy of Mavacamten has been extensively demonstrated in trials. In a global trial Olivotto et al., Mavacamten decreased the LVOT gradient while concurrently enhancing exercise capacity substantially, the New York Heart Association functional class (NYHA-FC), and overall health of the oHCM population [17]. Additionally, in the trial by Miland et al., Mavacamten proved to be a game-changer by significantly reducing the need for invasive SRT after therapy for 16 or 32 weeks who met the inclusion criteria for SRT among oHCM patients [18].

The regulatory approval for Mavacamten spans the US, Europe, and several other countries on five continents, specifically for adults experiencing symptomatic NYHA classes II–III oHCM. Mavacamten's action as a cardiac-specific myosin adenosine triphosphatase inhibitor results in the reversible inhibition of actin–myosin cross-bridging, consequently mitigating hypercontractility and enhancing myocardial energetics. In phase 2 open-label study, Mavacamten demonstrated excellent tolerability and markedly decreased post-exercise LVOT gradients in patients with oHCM [19]. In a phase 3 trial involving the Chinese oHCM population, Mavacamten significantly decreased LVOT gradient and enhanced cardiac structure, functional class, cardiac biomarkers, and health status compared to a placebo over 30 weeks. The safety profile aligns with the previous studies, affirming Mavacamten's efficacy and safety in Asian patients, including Chinese individuals, who often have a higher prevalence of poor CYP2C19 metabolizers and an average lower BMI than the worldwide population [20].

In this research, our aim is to perform an extensive meta-analysis by leveraging existing trials to assess both the efficacy and safety of Mavacamten in comparison with a placebo as a targeted approach for managing HCM.

## Methods

### Data sources and search strategy

This meta-analysis adhered to the guidelines outlined by PRISMA. Our approach to conducting a comprehensive systematic review and meta-analysis involved an exhaustive search of PubMed and the Cochrane Library for studies published up to September 20, 2023. By utilizing both of these reputable databases, our intention was to mitigate the potential for publication bias. To optimize our search strategy, we meticulously crafted a search string, thoughtfully combining various key terms such as “Hypertrophic cardiomyopathy,” “Mavacamten,” “New York Heart Association functional class,” “Septal reduction therapy,” “Atrial fibrillation,” and “adverse events.” Subsequently, articles meeting our criteria were manually retrieved and identified for further evaluation.

### Data extraction and quality assessment

We initiated the study selection process by first screening titles and abstracts to exclude studies that did not align with our predefined eligibility criteria. To prevent the inclusion of duplicate articles, we employed the EndNote Reference Library program. Upon identifying potential candidates, full-text articles were procured and subjected to a thorough examination to determine their suitability for inclusion in our meta-analysis. For the extraction of data, we prioritized consistency and precision. To achieve this, two authors, (HF and HSR), collaborated in the meticulous extraction of relevant information from each of the selected randomized controlled trials (RCTs). The data extracted encompassed a range of essential elements, including baseline characteristics, intervention particulars, and outcomes. In order to gauge the quality of the studies incorporated into our analysis, one of our authors, (MA), employed the Cochrane risk of bias tool for randomized trials (RoB 2). This rigorous assessment aimed to evaluate the potential risk of bias within each study, ensuring integrity of our findings.

### Outcomes

Primary outcomes in our analysis were (a): the number of patients meeting the primary endpoint as defined in each individual study. The primary endpoints for each trial are as follows:

EXPLORER-HCM [17]: The primary endpoint measure involved evaluating a combination of factors to gauge the clinical response at the 30-week mark in comparison with the baseline. This combination was defined as either a minimum increase of 1.5 mL/kg per minute or more in pVO_2_ along with at least one reduction in NYHA class, or an improvement of 3.0 mL/kg per minute or more in pVO_2_ without any deterioration in NYHA class.

MAVERICK-HCM [21]: The primary endpoint is characterized as follows: In Type 1, it is defined as an improvement from the baseline to week 16 of at least 1.5 mL/kg/min in pVO_2_ along with a reduction of 1 or more in NYHA class. In Type 2, it is defined as an improvement of at least 3.0 mL/kg/min in pVO_2_ with no deterioration in NYHA class, unless stated otherwise.

VALOR-HCM [18]: The primary endpoint was determined as a composite of either the decision to proceed with SRT or meeting the eligibility criteria for SRT in accordance with the 2011 American College of Cardiology (ACC) and American Heart Association (AHA) guidelines.

EXPLORER-CN [20]: The primary endpoint of interest was the alteration in Valsalva LVOT (left ventricular outflow tract) peak gradient from the starting point to week 30, as assessed using Doppler echocardiography.

Other primary outcomes included, (b) ≥ 1 New York Heart Association functional class improvement from baseline and (c) septal reduction therapy or eligibility for septal reduction therapy.

Secondary outcomes in our analysis included (a) ≥ 1 serious adverse event (SAE), (b) ≥ 1 treatment emergency adverse event (TEAE), (C) atrial fibrillation (AF), and (d) non-sustained ventricular tachycardia (NSVT).

### Statistical analysis

In conducting the statistical analysis for our meta-analysis, we utilized the Review Manager Software Package (Review Manager, Version 5.4.1, The Cochrane Collaboration, 2020). To assess the significance of differences between the Mavacamten and placebo groups, we calculated the risk ratio (RR) along with 95% confidence intervals (CIs). When dealing with studies that exhibited homogeneity in their results, we applied a fixed-effect model. Conversely, for studies that displayed significant heterogeneity, we employed a random-effect model. The choice of model was made based on the extent of observed heterogeneity, which was evaluated using the *I*^2^ statistic. *P* value below 0.05 was considered statistically significant.

## Results

### Studies selection

**Fig. 1:**
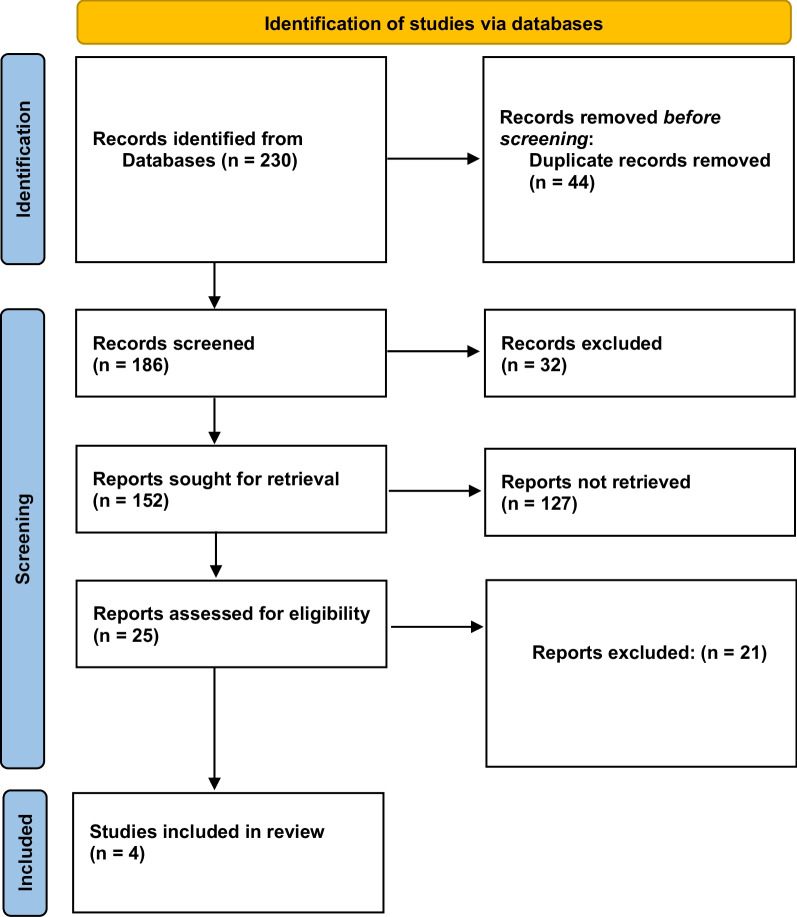
PRISMA flowchart

Shortlisting of studies is shown in Fig. 1. Our initial search retrieved 230 studies. Duplication of 44 records was found, which were then removed. Thirty-two studies were excluded for irrelevance. One hundred and fifty-two studies were selected for further assessment because of their relevance to the subject. After that, the exclusion of 127 studies was done as they were systematic reviews and non-RCTs. As a result, the final selection included four RCTs [17, 18, 20, 21] for meta-analysis.

### Baseline characteristics of the shortlisted trials

The four randomized controlled trials (RCTs) in question, namely, EXPLORER-HCM [17], MAVERICK-HCM [21], VALOR-HCM [18], and the recent EXPLORER-CN [20], involved 503 HCM diagnosed patients (273 were given Mavacamten and 230 received a placebo). In all the studies, average age of the patients exceeded 50 years, and approximately half of the participants were male [with percentages of 59.5% in EXPLORER-HCM [17], 39.6% in MAVERICK-HCM [21], 51% in VALOR-HCM [18], and 71.6% in EXPLORER-CN [20]]. The mean duration of follow-up was 23 weeks, with variations of 30 weeks in EXPLORER-HCM [17] and EXPLORER-CN [20] and 16 weeks in MAVERICK-HCM [21] and VALOR-HCM [18], EXPLORER-HCM, VALOR-HCM, and EXPLORER-CN [17, 18, 20] enrolled patients with oHCM, while MAVERICK-HCM [21] trial included patients with nHCM; due to the random allocation of patients to either the Mavacamten treatment group or the placebo group, most baseline characteristics were similar between the two groups. Table 1 contains details about the RCTs, while patients' baseline attributes are presented in Table 2.

**Table 1:** RCT characteristics of included investigations comparing Mavacamten treatment versus placebo in patients with HCM

Trial name	Country	Phase	Population					Design	
			Age (years)	Condition	Total (*n*)	Mavacamten (*n*)	Placebo (*n*)	Blinding	Treatment duration (weeks)
EXPLORER-HCM (NCT03470545) [17]	International	3	≥ 18	oHCM	251	123	128	Double-blinded	30
MAVERICK-HCM (NCT03442764) [21]	US	2	≥ 18	nHCM	59	40	19	Double-blinded	16
VALOR-HCM (NCT04349072) [18]	US	3	≥ 18	oHCM	112	56	56	Double-blinded	16
EXPLORER-CN (NCT05174416) [20]	China	3	≥ 18	oHCM	81	54	27	Double-blinded	30

*HCM* hypertrophic cardiomyopathy, *nHCM* non-obstructive hypertrophic cardiomyopathy, *oHCM* obstructive hypertrophic cardiomyopathy, and *RCT* randomized controlled trial

**Table 2:** Baseline clinical characteristics in Mavacamten treatment group versus placebo group

	EXPLORER-HCM (NCT03470545) [17]		MAVERICK-HCM (NCT03442764) [21]		VALOR-HCM (NCT04349072) [18]		EXPLORER-CN (NCT05174416) [20]	
	Mavacamten	Placebo	Mavacamten	Placebo	Mavacamten	Placebo	Mavacamten	Placebo
Age (years)	58.5	58.5	54	53.8	59.8	60.9	52.4	51
Male	54	65	47.5	31.6	51.8	50	41	17
BMI, kg m^−2^	29.7 (4.9)	29.2 (5.6)	29.3 (5.2)	31 (4.9)	29.3 (4.8)	31.9 (6.2)	25.2 (3.5)	26.1 (3.1)
HCM genetic testing perform	73	78	70	63.2	–	–	–	–
Pathogenic or likely pathogenic HCM gene variant	31	22	50	66.7	–	–	–	–
Medical history								
Family history of HCM	27	28	–	–	30.4	26.8	–	–
SRT	9	6	–	–	–	–	–	–
Diabetes mellitus	5	6	–	–	–	–	–	–
Dyslipidemia	22	30	–	–	–	–	4	0
Hypertension	46	41	–	–	64.3	60.7	3	0
Smoking	–	–	–	–	–	–	–	–
Obesity	12	11	–	–	–	–	–	–
Coronary artery disease	10	5	–	–	–	–	–	–
ICD	22	23	–	–	16.1	17.9	–	–
Atrial fibrillation	10	18	–	–	19.6	14.3	2	0
Chronic lung disease	16	11	–	–	–	–	–	–
Background HCM therapy								
Beta-blocker	76	74	62.5	63.2	46.4	44.6	48	24
Calcium channel blocker	20	13	25	15.8	12.5	17.9	4	2
NYHA-FC II	72	74	82.5	68.4	7.1	7.1	44	18
NYHC-FC III	28	26	17.5	31.6	92.9	92.9	10	9
pVO_2_, ml/kg/min	18.9 (14.9)	19.9 (4.9)	20.4 (6)	17.9 (5.1)	–	–	–	–
Echocardiac parameters								
LVEF, %	74 (6)	74 (6)	68.7 (5.5)	66.4 (7.7)	67.9 (3.7)	68.3 (3.2)	77.8 (6.9)	77 (6.7)
Maximum left ventricular wall thickness, mm	20 (4)	20 (3)	20.6 (4.0)	18.8 (3.5)	–	–	22.9 (4.9)	24.3 (6.4)
LVOT gradient, rest, mm Hg	52 (29)	51 (32)	–	–	51.2 (31.4)	46.3 (30.5)	74.6 (35.1)	73.4 (32.2)
LVOT gradient, Valsalva, mm Hg	72 (32)	74 (32)	–	–	75.3 (30.8)	76.2 (29.9)	106.8 (43.2)	99.8 (41.1)
LVOT gradient post-exercise, mm Hg	86 (34)	84 (36)	–	–	82.5 (34.7)	85.2 (37)	–	–
Left atrial volume index, ml/m^2^	40 (12)	41 (14)	37.3 (13)	40.8 (15.2)	41.3 (16.5)	40.9 (15.2)	43.3 (12.1)	47.5 (14.7)
Left atrial diameter, mm	42 (5)	42 (6)	–	–	–	–	–	–

Data are presented as % or mean (standard deviation)

*BMI* body mass index, *HCM* hypertrophic cardiomyopathy, *ICD* implantable cardioverter defibrillator, *LVEF* left ventricular ejection fraction, *LVOT* left ventricular outflow tract, *NYHA-FC* New York Heart Association functional class, *pVO*_*2*_ peak oxygen consumption, *SRT* septal reduction therapy, and – no information available

### Quality assessment

**Fig. 2:**
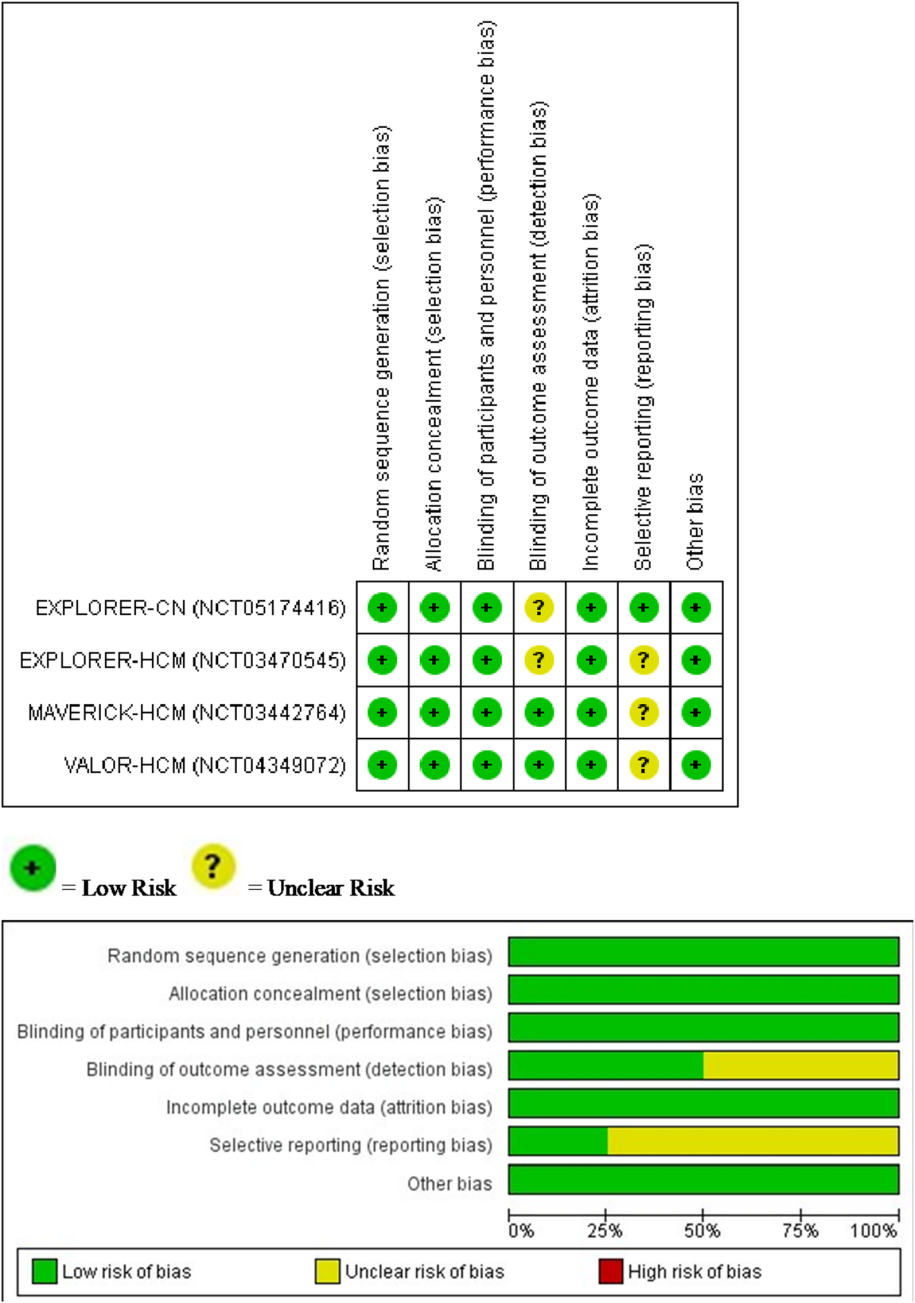
Quality judgment about each risk of bias item

The Cochrane risk of bias 2 tool was used to assess the studies, and the findings are presented in Fig. 2. All our studies were considered to have minimal risk of bias, indicating a high level of reliability.

### Cardiac outcomes

**Fig. 3:**
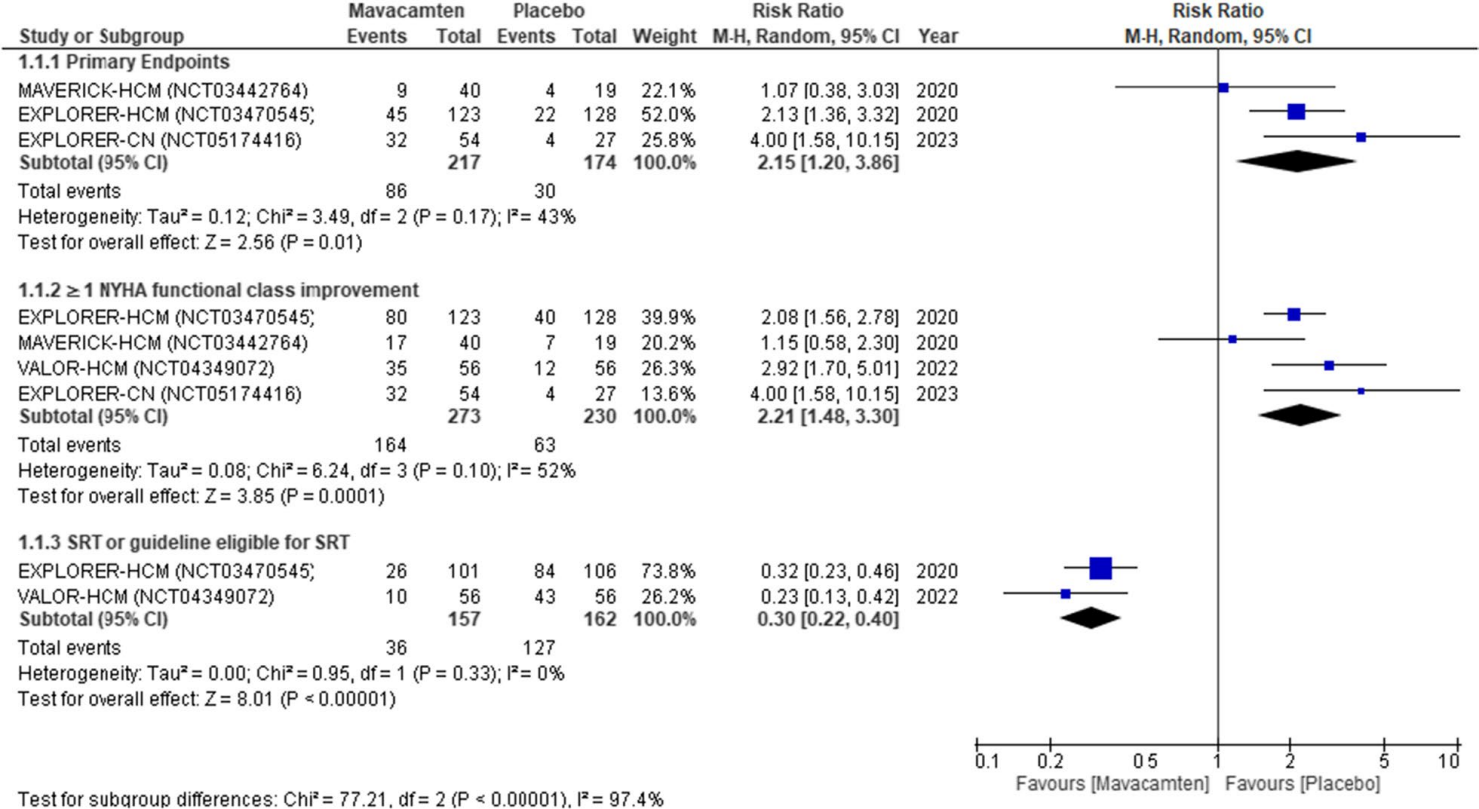
Forest plot for cardiac outcomes

Mavacamten demonstrated a substantial increase in the primary endpoint achievement (115% increase) with a relative risk (RR) of 2.15 (95% CI 1.20–3.86; *P* = 0.01, Fig. 3 (1.1.1)), despite moderate heterogeneity (I^2^ = 43%). Additionally, there was a noteworthy improvement of ≥ 1 New York Heart Association functional class improvement (121% increase) with an RR of 2.21 (95% CI 1.48–3.3; *P* = 0.0001, Fig. 3 (1.1.2)), although heterogeneity remained relatively high (*I*^2^ = 52%). We conducted a sensitivity analysis by excluding the MAVERICK-HCM [21], which still yielded significant results (146% increase) with an RR of 2.46 (95% CI 1.77–3.42; *P* < 0.0001) and lower heterogeneity (*I*^2^ = 25%). Mavacamten also exhibited substantially lower rates of SRT or eligibility for SRT (70% lower rates) compared to those receiving placebo, with an RR of 0.30 (95% CI 0.22–0.40; *P* < 0.00001, Fig. 3 (1.1.3)), and minimal heterogeneity (*I*^2^ = 0%).

### Adverse outcomes

**Fig. 4:**
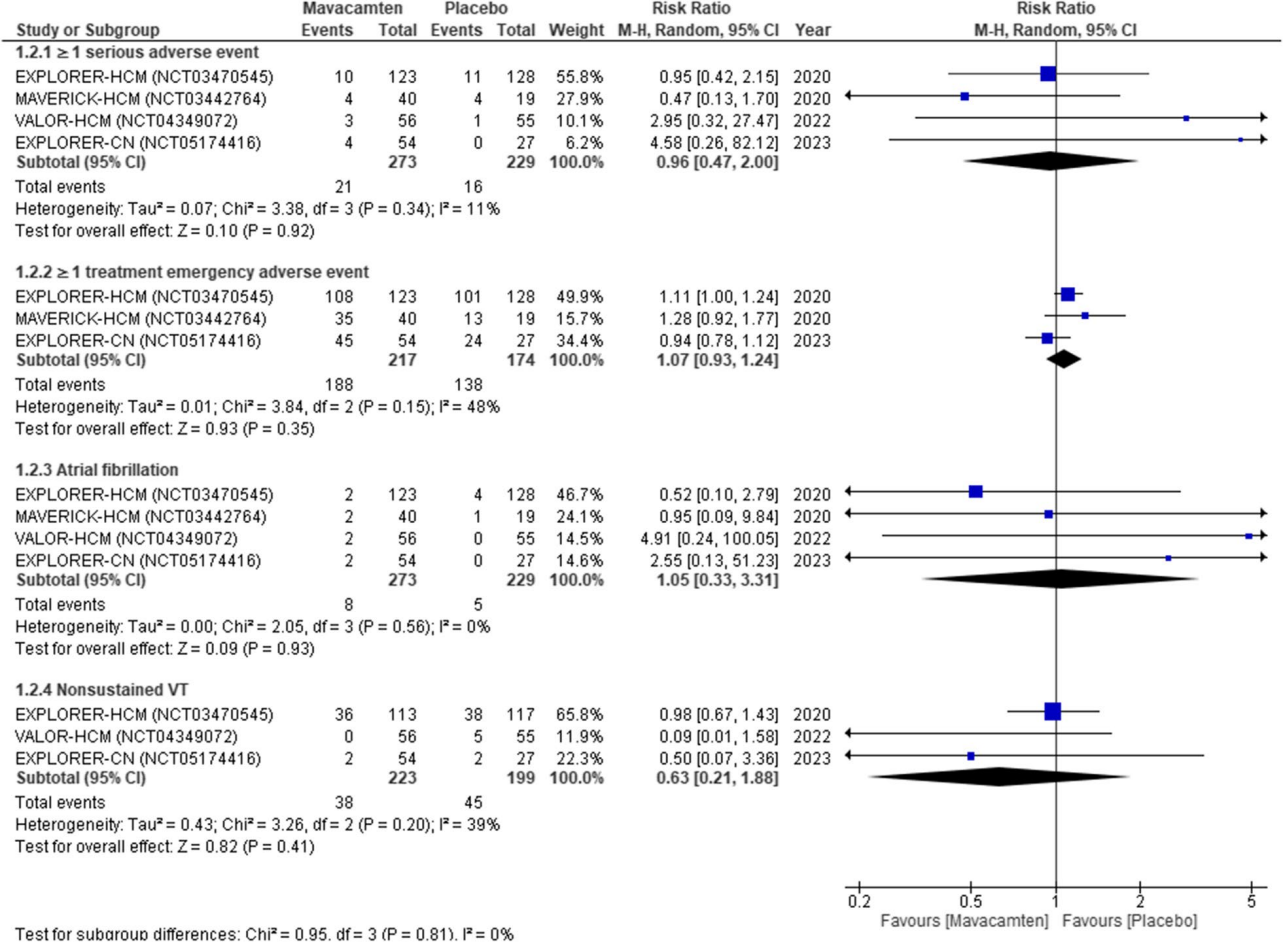
Forest plot for adverse outcomes

There were no statistically significant differences observed between both groups in terms of ≥ 1 SAE with a relative risk (RR) of 0.96 (95% CI 0.47–2.00; *P* = 0.92, Fig. 4 (1.2.1)), and heterogeneity remained low at *I*^2^ = 11%. Similarly, there were no significant disparities in ≥ 1 TEAE with an RR of 1.07 (95% CI 0.93–1.24; *P* = 0.35, Fig. 4(1.2.2)), albeit with moderate heterogeneity at *I*^2^ = 48%. The incidence of AF also showed no significant difference, with an RR of 1.05 (95% CI 0.33–3.31; *P* = 0.93, Fig. 4(1.2.3)) and no heterogeneity (*I*^2^ = 0%). Lastly, NSVT rates exhibited no statistically significant divergence, with an RR of 0.63 (95% CI 0.21–1.88; *P* = 0.41, Fig. 4(1.2.4)) and moderate heterogeneity at *I*^2^ = 39%.

## Discussion

This meta-analysis, involving a patient population of 503, aimed to compare the safety and efficacy of Mavacamten with placebo in recent HCM patients. The study's results indicated a significant association between Mavacamten and achieving a primary endpoint, ≥ 1 New York Heart Association functional class improvement compared to placebo. However, it showcased a non-significant association between both groups regarding ≥ 1 SAE, ≥ 1 TEAE, the incidence of AF, and NSVT rates.

Our analysis revealed a significant association between achieving the primary endpoint and administering Mavacamten instead of a placebo. Mavacamten, effectively reduces excessive myocardial contractility, resulting in enhanced left ventricular diastolic filling, alleviation of LVOT, and improved ventricular lusitropy [22]. This resultant enhancement in the left ventricular compliance reduces symptom severity experienced by HCM patients, both at rest and during exertion. These positive outcomes are reflected in our analysis of the primary composite endpoint. Furthermore, the moderate heterogeneity observed in this outcome indicates that the results maintain a reasonable degree of consistency across the various studies.

In contrast with placebo, the analysis demonstrated a significant difference between Mavacamten and ≥ 1 NYHA-F class. The recent EXPLORER-CN [20], involving 81 participants (Mavacamten *n* = 54, placebo *n* = 27), reported that patients in the Mavacamten group had increased improvement in ≥ 1 NYHA-FC-II (82% vs. 68%). Compared to placebo, Mavacamten appears to cause a significant reduction in heart failure symptoms as measured by the NYHA-FC in HCM patients. This is due to its pharmacological mechanism of action, acting as a direct target inhibitor of cardiac myosin, decreasing myosin binding with actin filament, and the excessive contractility that is a feature of HCM [23]. In EXPLORER-HCM [17], too, Mavacamten was superior to placebo in improving patient-centered outcomes. Moreover, the moderate to high heterogeneity observed in this outcome showcases that the results are sufficiently consistent between the studies. However, MAVERICK-HCM [21] acknowledged that their study had relatively small size to notice clinical improvements by pVO_2_ or NYHA-FC class, urging the need for further randomized control trials to be conducted to draw a conclusive understanding [21]. Lastly, the effect size (RR increased from the initial value to 2.46 when the MAVERICK-HCM [21] was excluded) and heterogeneity (*I*^2^ fell from 52 to 25%) significantly changed. This suggests that the MAVERICK-HCM [21] study, having significant differences from other studies pooled, had a major impact on the overall outcomes. Its absence resulted in less heterogeneity among the other trials and a more substantial observed effect.

In oHCM population, Mavacamten was associated with lesser number of SRT and its eligibility compared to placebo, as exhibited by its significant relation. This suggests that Mavacamten has therapeutic benefits that mitigate the progression of oHCM and slow the development of oHCM to the point where invasive therapies like SRT are no longer required. Furthermore, no heterogeneity is observed between the studies. This strengthens the study results and suggests that the findings are similar across investigations, which is advantageous when analyzing the combined findings from meta-analyses.

Our analysis showed no significant associations between Mavacamten and placebo regarding the rates of ≥ 1 SAE and NSVT. A lower risk ratio for ≥ 1 SAE and NSVT indicates that Mavacamten showed decreased risk of these adverse events compared to placebo, underscoring its safety profile as a therapeutic drug. Furthermore, according to a report on Mavacamten-controlled HCM, patients with symptomatic oHCM, when treated with Mavacamten for a median of 62 weeks, maintained the same safety response observed during the first 30 weeks of the drug's pivotal trial [24]. However, the non-significant disparity suggests that these findings could be due to other factors, such as variability in dosage, duration of use, and the characteristics of the study population. Additionally, its insignificant relation with NSVT rates suggests that early detection and treatment of any rhythm abnormalities might be accomplished with routine monitoring, such as through ECGs, which would lessen the likelihood that NSVT would progress. Despite the non-significant association of Mavacamten with NSVT, nullifying its safety, the VALOR-HCM [18] study resulted in no non-sustained ventricular tachycardia found in the Mavacamten group as opposed to 9.1% in the placebo group [25].

Lastly, there was no significant relation between the two groups causing TEAE or increasing the incidence of atrial fibrillation. However, the risk of atrial fibrillation and ≥ 1 TEAE is 5% and 7% higher in the Mavacamten group compared to the placebo group. Notably, the recent EXPLORER-CN [20] study found atrial fibrillation as an adverse outcome in only two out of 54 patients in the Mavacamten group. Still, the pooled analysis presented in Fig. 4 did not yield a significant result. Additionally, an integrated analysis of data from already conducted HCM trials indicates that Mavacamten is generally safe and well-tolerated across various dosages, irrespective of obstruction presence [25]. These results emphasize the need for more extensive and well-powered RCTs to delve deeper into the mechanisms and potential confounding factors linked with Mavacamten-related adverse events.

### Limitations

Our study exhibited several significant limitations. Firstly, the study only includes four RCTs, observed by a small sample size and fewer documented adverse events. Secondly, the analysis combines information from both oHCM and nHCM, which could lead to contradictions. Even though they are both HCM subtypes, their treatments and clinical outcomes vary. Thirdly, on average, the included studies' follow-up periods lasted roughly 23 weeks. To completely comprehend Mavacamten's long-term effects, studies with a long follow-up period are necessary. Fourthly, our research's quality is organically tied to the caliber of the studies it incorporates and is constrained by their constraints, just like any meta-analysis [26].

## Conclusions

Our findings suggest that comparatively, Mavacamten does exhibit therapeutic effects on hypertrophic cardiomyopathy patients, as shown by its notable association with improved primary composite endpoint, ≥ 1 NYHA-FC class improvement, and lower rate of eligibility for SRT. Our study, however, did not find a significant difference between Mavacamten and placebo and its impact on adverse events such as ≥ 1 SAE, ≥ 1 TEAE, the incidence of AF, and NSVT rates. These findings highlight the need for future research with larger sample sizes, more extended follow-up periods, and comprehensive assessment of various contributing factors that may lead to these outcomes, giving further insights into the safety and efficacy of Mavacamten administered in hypertrophic cardiomyopathy patients.

## Data Availability

Data available within the article. The authors confirm that the data supporting the findings of this study are available within the article.

## Abbreviations

HCM: Hypertrophic cardiomyopathy
LV: Left ventricular
oHCM: Obstructive HCM
LVOT: Left ventricular outflow tract
nHCM: Non-obstructive HCM
SRT: Septal reduction therapy
NYHA-FC: New York Heart Association functional class
RCTs: Randomized controlled trials
SAE: Serious adverse event
TEAE: Treatment emergency adverse event
AF: Atrial fibrillation
NSVT: Non-sustained ventricular tachycardia
RR: Risk ratio
CIs: Confidence intervals
*I*^2^: *I*-squared
RevMan: Cochrane Review Manager
PRISMA: Preferred Reporting Items for Systematic Reviews and Meta-Analyses
